# Highly efficient capture approach for the identification of diverse inherited retinal disorders

**DOI:** 10.1038/s41525-023-00388-3

**Published:** 2024-01-09

**Authors:** Hsiao-Jung Kao, Ting-Yi Lin, Feng-Jen Hsieh, Jia-Ying Chien, Erh-Chan Yeh, Wan-Jia Lin, Yi-Hua Chen, Kai-Hsuan Ding, Yu Yang, Sheng-Chu Chi, Ping-Hsing Tsai, Chih-Chien Hsu, De-Kuang Hwang, Hsien-Yang Tsai, Mei-Ling Peng, Shi-Huang Lee, Siu-Fung Chau, Chen Yu Chen, Wai-Man Cheang, Shih-Jen Chen, Pui-Yan Kwok, Shih-Hwa Chiou, Mei-Yeh Jade Lu, Shun-Ping Huang

**Affiliations:** 1 Institute of Biomedical Sciences, Academia Sinica, Taipei, 115201 Taiwan; 2 Doctoral Degree Program of Translational Medicine, National Yang Ming Chiao Tung University and Academia Sinica, Taipei, 115201 Taiwan; 3 Institute of Medical Sciences, Tzu Chi University, Hualien, 970374 Taiwan; 4 Biodiversity Research Center, Academia Sinica, Taipei, 115201 Taiwan; 5 Department of Medical Research, Taipei Veterans General Hospital, Taipei, 112201 Taiwan; 6 Department of Ophthalmology, Taipei Veterans General Hospital, Taipei, 112201 Taiwan; 7 Institute of Pharmacology, National Yang Ming Chiao Tung University, Taipei, 112304 Taiwan; 8 School of Medicine, National Yang Ming Chiao Tung University, Taipei, 112304 Taiwan; 9 Department of Ophthalmology, Taichung Tzu Chi Hospital, Taichung, 427003 Taiwan; 10 Institute for Human Genetics, Cardiovascular Research Institute, and Department of Dermatology, University of California, San Francisco, CA USA; 11 Genomic Research Center, Academia Sinica, Taipei, 115201 Taiwan; 12 Department of Molecular Biology and Human Genetics, Tzu Chi University, Hualien, 970374 Taiwan

**Keywords:** Genetics research, Sequencing

## Abstract

Our study presents a 319-gene panel targeting inherited retinal dystrophy (IRD) genes. Through a multi-center retrospective cohort study, we validated the assay’s effectiveness and clinical utility and characterized the mutation spectrum of Taiwanese IRD patients. Between January 2018 and May 2022, 493 patients in 425 unrelated families, all initially suspected of having IRD without prior genetic diagnoses, underwent detailed ophthalmic and physical examinations (with extra-ocular features recorded) and genetic testing with our customized panel. Disease-causing variants were identified by segregation analysis and clinical interpretation, with validation via Sanger sequencing. We achieved a read depth of >200× for 94.2% of the targeted 1.2 Mb region. 68.5% (291/425) of the probands received molecular diagnoses, with 53.9% (229/425) resolved cases. Retinitis pigmentosa (RP) is the most prevalent initial clinical impression (64.2%), and 90.8% of the cohort have the five most prevalent phenotypes (RP, cone-rod syndrome, Usher’s syndrome, Leber’s congenital amaurosis, Bietti crystalline dystrophy). The most commonly mutated genes of probands that received molecular diagnosis are *USH2A* (13.7% of the cohort), *EYS* (11.3%), *CYP4V2* (4.8%), *ABCA4* (4.5%), *RPGR* (3.4%), and *RP1* (3.1%), collectively accounted for 40.8% of diagnoses. We identify 87 unique unreported variants previously not associated with IRD and refine clinical diagnoses for 21 patients (7.22% of positive cases). We developed a customized gene panel and tested it on the largest Taiwanese cohort, showing that it provides excellent coverage for diverse IRD phenotypes.

## Introduction

Molecular diagnosis of rare diseases is challenging, and few treatments for genetic disorders are currently available^1,2^. Globally, 2.7 billion individuals (36% of the population) carry gene mutations responsible for autosomal recessive inherited retinal dystrophy (AR-IRD), and 5.5 million are afflicted with these mostly untreatable disorders^3^. Recent advances in DNA sequencing technologies and gene therapies have improved diagnostic yield and increased treatment options^4^. However, clinical translation needs to catch up to scientific discovery. For mutations in more than 300 genes associated with IRD identified to date, only mutations in fifteen genes (*ABCA4*, *CEP290*, *CHM/REP1*, *CYP4V2*, *GUCY2D*, *MERTK*, *NR2E3*, *PDE6A*, *PDE6B*, *RHO*, *RPE65*, *RLBP1*, *RPGR*, *RS1*, *USH2A*) are currently investigated for therapy. Clinical trials based on the mutations in these genes have been studied for seven IRDs (Enhanced S-cone syndrome (ESCS), Leber’s Congenital Amaurosis (LCA), Rod-Cone dystrophy (RCD), Retinitis Pigmentosa (RP), Stargardt’s dystrophy (SD), Usher’s syndrome (US) (Supplementary Table 1). In 2017, Luxturna (voretigene neparvovec-rzyl) became the first FDA-approved gene therapy in the USA, targeting biallelic mutations in *RPE65* for LCA2, and remains the only approved IRD gene therapy available. As the vast majority of this group of disorders is untreatable, most patients progress to early blindness. Establishing the molecular diagnosis of IRDs is the first step in identifying possible therapeutic targets, and characterizing the mutational spectrum of IRD in the population can help prioritize efforts to develop treatments.

IRDs are genetically heterogeneous diseases that manifest a spectrum of phenotypes. IRDs exist as syndromic and non-syndromic forms where the former is associated with extra-ocular features, and the latter is confined to the eye. It has been estimated that up to 30% of IRD are syndromic, so ocular manifestations and molecular diagnosis obtained before the development of extra-ocular features may aid in timely diagnosis and management^5–7^. This paper presents the highly efficient molecular diagnostic approach for IRDs based on next-generation sequencing of a gene panel of 319 IRD-associated genes. We tested this approach on 425 patients and critical family members, representing the largest Taiwanese IRD cohort to date, and obtained a diagnostic yield of 68.5% of the probands received molecular diagnoses, and 53.9% of those consisted of solved diagnoses. Our results established the Taiwanese IRD genetic landscape and demonstrated that gene panel sequencing could be a cost-effective and highly efficient diagnostic method for IRD in both research and clinical settings.

## Results

### Cohort characteristics

This study identified 493 individuals, including 425 probands, with clinically suspected IRD (Table 1) (Supplementary Data 1: Phenodata). Age of onset was known in 365/425 probands, and the mean proband onset age was 25.0 (range, 0.08-74.0). Detailed clinical information per phenotype is delineated in Table 2. A tenth of the probands (46/425, 10.8%) had extra-ocular signs and symptoms consistent with syndromic IRDs, including Usher (38/425, 8.9%), Stickler (5/425, 1.2%), Alstrom (1/425, 0.002%), and Bardet-Biedl (2/425, 0.005%) syndromes. The subjects were grouped according to their initial clinical diagnosis. The breakdown of the 425 study probands among high-order diagnostic categories is shown in Table 2. RP is the most prevalent IRD (273/425, 64.2%), with the five most common conditions (RP, CRD, US, LCA, and BCD) accounting for 90.8% of the cohort (387/425).

**Table 1:** Diagnostic yield.

*Molecular diagnosis of proband*		
Probands with molecular diagnosis	291/425	(68.5%)
Probands with ACMG molecular diagnosis	240/425	(56.5%)
Molecular diagnosis confirmed clinical diagnosis	270/291	(92.8%)
Molecular diagnosis led to alternate clinical diagnosis	21/291	(7.2%)
*Family history*		
Cases with a family history of IRD	112/129	(88.9%)
Sporadic cases	179/296	(60.5%)
*IRD category*		
Syndromic IRD	37/46	(80.4%)^a^
Non-syndromic IRD	254/379	(67.0%)^a^

The table depicts the diagnostic yield of probands and the proportions of molecularly diagnosed patients receiving an alternative clinical diagnosis. The diagnostic yield is broken down into different groups, such as family history and IRD category. Proband phenotype uses initial diagnosis; for the unsolved proband, the final diagnosis is the same as the initial diagnosis.

^a^Diagnostic yield per phenotype category: the numerator denotes the number of probands with a molecular diagnosis, and the denominator denotes the number of probands of the phenotype category.

**Table 2:** Diagnostic yield per initial diagnosis of the proband.

Phenotype	Female	(%)	Solved rate	(%)	Receive molecular diagnosis (%)		Sporadic	(%)	Age	(range)	Onset age	(range)	VA (OD)	(Range)	VA (OS)	(range)
AS	0/1	0.00	0/1	0.00	0/1	0.00	1/1	100.0	57.8	(57.8, 57.8)	48.0	(48.0, 48.0)	0.2	(0.16, 0.16)	2.4	(2.40, 2.40)
BBS	1/2	50.00	1/2	50.00	1/2	50.0	1/2	50.0	47.8	(29.5, 66.2)	19.0	(3.00, 35.0)	2.4	(2.10, 2.70)	2.6	(2.40, 2.70)
BCD	9/13	69.23	12/13	92.31	12/13	92.3	10/13	76.9	57.2	(40.0, 76.9)	32.8	(20.0, 45.0)	1.1	(0.00, 2.70)	1.1	(0.22, 2.70)
BVMD	0/1	0.00	1/1	100.00	1/1	100.0	0/1	0.00	62.4	(62.4, 62.4)	40.0	(40.0, 40.0)	1.8	(1.78, 1.78)	1.3	(1.30, 1.30)
CD	2/2	100.00	1/2	50.00	2/2	100.0	2/2	100.0	51.5	(35.5, 67.4)	31.5	(3.00, 60.0)	0.9	(0.22, 1.52)	1.5	(1.30, 1.70)
Choroideremia	1/9	11.11	7/9	77.78	7/9	77.8	2/9	22.2	47.8	(19.9, 66.5)	21.9	(1.00, 55.0)	0.8	(0.00, 2.40)	0.8	(0.00, 2.70)
CRD	17/43	39.53	23/43	53.48	30/43	69.8	29/43	67.4	50.6	(17.9, 72.7)	26.8	(3.00, 65.0)	1.5	(0.00, 3.00)	1.3	(0.00, 3.00)
LCA	15/19	78.95	16/19	84.21	17/19	89.5	15/19	78.9	34.4	(1.83, 82.3)	9.41	(0.50, 46.0)	1.4	(0.00, 2.70)	1.4	(0.00, 2.70)
MD	5/9	55.56	4/9	44.44	7/9	77.8	6/9	66.7	58.4	(26.0, 74.1)	38.0	(15.0, 63.0)	0.5	(0.00, 1.30)	1.0	(0.00, 2.40)
Optic atrophy	1/1	100.00	1/1	100.00	1/1	100.0	1/1	100.0	12.2	(12.2, 12.2)	3.00	(3.00, 3.00)	0.8	(0.80, 0.80)	0.8	(0.80, 0.80)
RP	136/273	49.82	128/273	46.89	168/273	61.5	193/273	70.7	50.9	(0.00, 96.4)	25.8	(0.25, 74.0)	1.1	(0.00, 3.00)	1.1	(0.00, 3.00)
RPA	1/2	50.00	2/2	100.00	2/2	100.0	1/2	50.0	37.6	(21.6, 53.6)	16.5	(3.00, 30.0)	0.7	(0.10, 1.30)	1.4	(1.00, 1.70)
RS	0/2	0.00	2/2	100.00	2/2	100.0	0/2	0.00	19.3	(15.3, 23.3)	7.00	(7.00, 7.00)	0.5	(0.52, 0.52)	0.5	(0.52, 0.52)
SD	2/5	40.00	5/5	100.00	5/5	100.0	5/5	100.0	37.1	(25.0, 60.2)	22.0	(6.00, 55.0)	1.3	(0.22, 2.10)	1.5	(0.52, 2.10)
SS	2/5	40.00	3/5	60.00	5/5	100.0	4/5	80.0	52.3	(4.25, 73.6)	32.9	(0.25, 50.0)	1.4	(0.16, 2.70)	1.7	(0.70, 3.00)
US	19/38	50.00	24/38	63.16	31/38	81.6	26/38	68.4	44.6	(0.00, 83.3)	23.8	(0.08, 60.0)	1.0	(0.00, 3.00)	1.0	(0.00, 3.00)
Total	211/425	49.65	229/425	53.88	291/425	68.5	296/425	69.6	49.5	(0.00, 96.4)	25.0	(0.08, 74.0)	1.1	(0.00, 3.00)	1.1	(0.00, 3.00)

The table shows the clinical demographic and diagnostic yield of each IRD phenotype. Phenotype abbreviations are listed in the supplementary table. Uncertain denotes phenotypes that ophthalmologists are unable to confirm the diagnosis.

*AS* Altrom’s syndrome, *BBS* Bardet Biedl’s syndrome, *BCD* Bietti’s crystalline dystrophy, *BVMD* best vitelliform macular dystrophy, *CD* cone dystrophy, *CRD* cone-rod dystrophy, *LCA* Leber’s congenital amaurosis, *MD* macular degeneration, *RP* retinitis pigmentosa, *RPA* retinitis punctata albescens, *RS* retinoschisis, *SD* Stargardt’s dystrophy, *SS* Stickler’s syndrome, *US* Usher’s syndrome.

### Diagnostic yield and genetic findings

We developed a high-throughput sequencing panel test and achieved over 200X coverage of 94.2% of the 1.20 Mb target region (Supplementary Data 2). We sequenced 782 subjects in total, including 425 probands, and made molecular diagnoses for 68.5% (291/425) of the probands (Supplementary Data 3: GenoData). The diagnostic yield of cases with a positive family history of IRD (88.9%, 112/129) is notably higher than that for sporadic cases (60.5%, 179/296). In addition, syndromic patients have a higher diagnostic rate (80.4%) compared to that for non-syndromic cases (60.5%) (Table 1). Overall, our approach achieved a high diagnostic yield for all IRD subtypes (Table 2**)** in patients of all age groups (Supplementary Table 3). Furthermore, among the 291 probands with positive molecular diagnoses, the clinical diagnoses were confirmed in 92.8% of the cases, while the molecular diagnoses in 7.22% of the cases led to alternate clinical diagnoses. (Table 1).

**Fig. 1:**
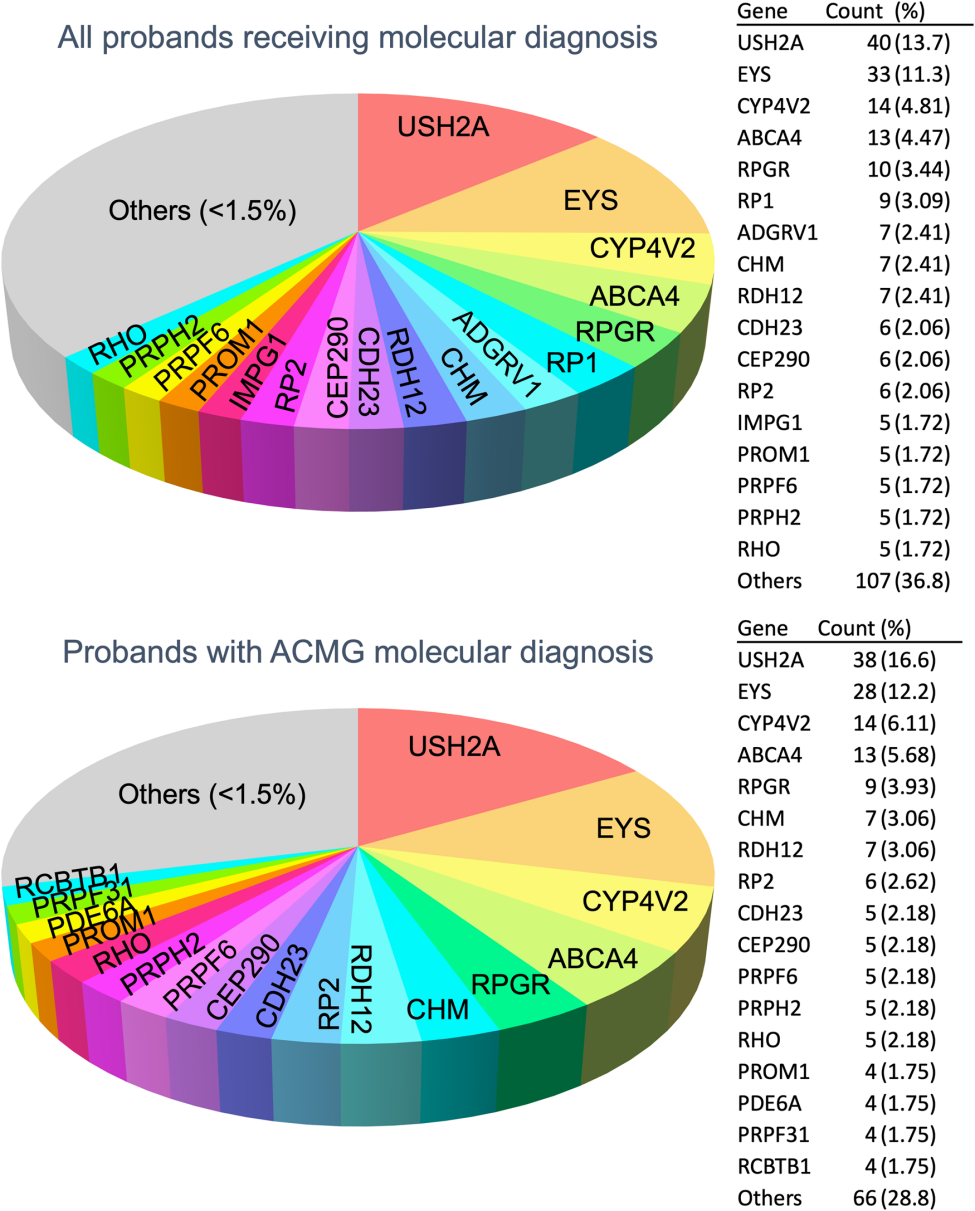
Genetic landscape. Pie chart showing the distribution of mutated genes in the 293 probands who received a molecular diagnosis after HRD panel genetic testing. Others denote accumulation of genes with <1.5% contribution.

Collectively, 568 variants (457 reported and 111 previously unreported) in 87 unique genes were identified (Table 3) (Supplementary Data 4). Mutations in 5 genes account for 40.8% (119/291) of the molecular diagnoses made: *USH2A* (40, 13.7%), *EYS* (33, 11.3%), *CYP4V2* (15, 4.8%), *ABCA4* (13, 4.5%), *RPGR* (9, 3.4%), and *RP1* (9, 3.1%). Almost half of the positive cases (49.8%) are due to mutations found in the top 10 genes: the five genes listed above plus *RDH12* (7, 2.4%), *CHM* (7, 2.4%), *ADGRV1* (7, 2.4%), *RP2* (6, 2.1%) (Fig. 1).

**Table 3:** Previously unreported variants identified.

Variant type	Probands with ACMG molecular diagnosis		All probands with molecular diagnosis	
Missense	32/243	(13.2%)	72/332	(21.7%)
In-frame indel	18/92	(19.6%)	19/97	(19.6%)
Non-frameshift indel	5/11	(45.5%)	5/12	(41.7%)
Stop gain/ loss	9/74	(12.2%)	11/79	(13.9%)
Splice-site	4/47	(8.51%)	4/48	(8.33%)
Total	68/467	(14.6%)	111/568	(19.5%)

The table shows the number of variants and proportions previously unreported of each variant type identified in probands with molecular diagnosis (*n* = 291) and those with molecular diagnosis based on strict ACMG criteria (*n* = 229). Homozygous variants are counted twice as allele occurrence.

**Fig. 2:**
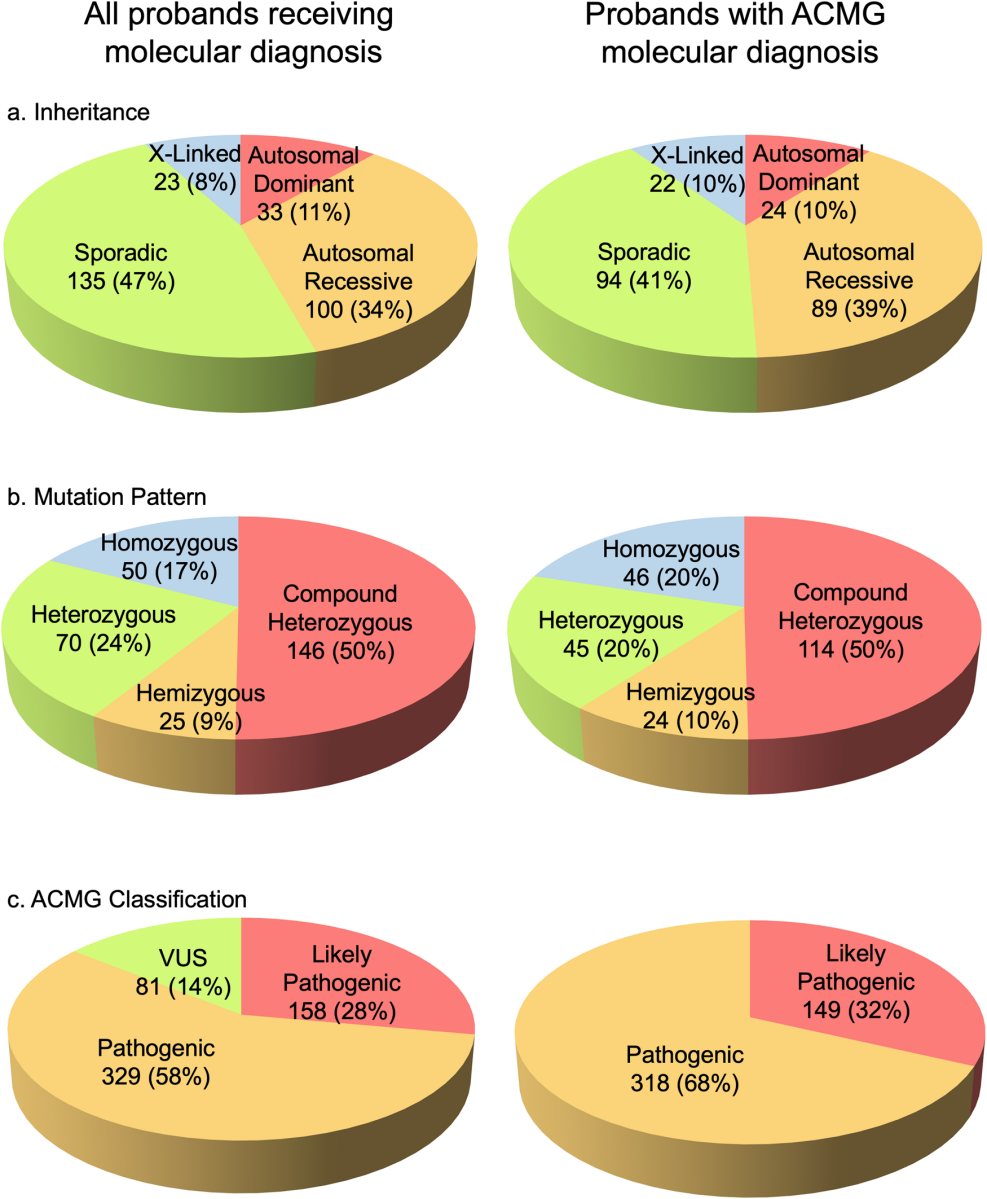
Genetic characteristics. Pie chart showing the distribution of mutated gene characteristics in the 293 probands. Genotype and Inheritance are based on probands (293). Mutation characteristics and ACMG pathogenicity are variant-based such that they represent a total number of variants in the cohort of 293 probands (a total of 512 variants; homozygous variants are duplicated as alleles in the number of occurrences).

Evaluating the mode of inheritance for all probands by pedigree (*n* = 425), we found that two-thirds of the probands have no family history of IRD (sporadic cases, 61.6%) (Table 4). We next studied the genotype and mode of inheritance for 291 positive cases. About half of the cases are sporadic (46.4%), 34.4% are of autosomal recessive inheritance, 11.3% are autosomal dominant, and 7.9% are X-linked recessive (Fig. 2a). Half of the positive cases (50.3%) have compound heterozygous mutation genotypes (Fig. 2b). Evaluating the ACMG pathogenicity classification of the 568 variants identified in the cohort, we found that 57.9% are pathogenic, 27.8% are likely pathogenic, and 14.3% are variants of unknown significance (Fig. 2c). Among the variants found, 111 variants have not been reported previously in databases associated with IRD (Table 3). Detailed genetic information on previously unreported variants is shown in Supplementary Data 3.

**Table 4:** Pedigree-based inheritance.

Inheritance	All proband	(%)	Probands with molecular diagnosis (%)		Probands with ACMG molecular diagnosis	(%)
AD	35	8.20	33	11.3	24	10.5
AR	103	24.2	100	34.4	89	38.9
S	262	61.6	135	46.4	94	41.0
XL	25	5.90	23	7.90	22	9.60
Grand Total	425	100.0	291	100.0	240	100.0

## Discussion

In this study, we custom-designed a high-quality target capture probe set for NGS of a panel of 319 IRD-associated genes and tested the IRD panel sequencing approach on the largest Taiwanese IRD cohort to date. The IRD gene panel design is optimized for coverage of as many IRD genes as possible, and the sequencing protocol ensures that very high read depth is achieved while we scan the samples in batches of 200 samples in one experiment. The result is a highly efficient process with uniformly high depth coverage (>200×) for the target region, minimal sample failures, and much higher diagnostic yield for a wide range of IRDs than most rare genetic disease sequencing studies^8–16^. In the process, we also identified 111 previously unreported causal variants that could be used for therapeutic target development. In addition, the mutation spectrum and heterogeneity in our IRD cohort are significantly different from those in cohorts of other studies and ancestries. We found that our IRD landscape follows that of East Asia, with top genes consisting of *USH2A*, *EYS*, and *CYP4V2*, and not *ABCA4*, which is mainly found in European cohorts (Supplementary Fig. 2, Supplementary Table 4)^9,13^.

Visual impairment in children is challenging to diagnose in the early stages as IRD is complicated by complex, ambiguous phenotypes, hampering timely clinical diagnosis. Although IRD is considered a pediatric genetic disease, the patients’ mean onset age is 25.0. Moreover, 61.6% reported a negative family history, implying a high carrier rate in Taiwan with asymptomatic parents or underdiagnosed family members (Table 2). Genetic testing provides an opportunity to confirm or refine clinical diagnosis, guide disease management, inform prognosis, and assist in family planning^17^. Increased access to testing may make a difference in how patients interpret, adapt to, and experience their condition and are informed as gene therapies become available. Where genetic mutations still present with no cure, genetic results allow the family to prepare and plan for the future to support their child as required and reduce psychosocial burden. The IRD panel we designed provides a high success rate in diagnosis for patients regardless of their family history, phenotype, disease status, gender, and age. With a high diagnostic yield for diverse IRD subtypes, the IRD sequencing panel we designed is useful for early genetic testing and routine implementation in the clinic for IRD patients.

## Methods

### Patient enrollment and DNA preparation

This study was approved by the Institutional Review Board (IRB) of Academia Sinica (AS-IRB01-21064(N)), Taipei Veterans General Hospital (TVGH, 2021-04-009A), and Tzu Chi University (TCU, REC107-24) and adheres to the tenets of the Declaration of Helsinki. Written informed consent was obtained from all participants and their guardians if they were under legal age. Four hundred ninety-three patients, including 425 probands aged 0–96 years with suspected IRD and no previous genetic diagnosis, visited the Department of Ophthalmology at the TVGH and TCU between January 2018 and May 2022. DNA from 289 unaffected family members was also analyzed (total participants 782). Age and symptom onset, family history, gender, and best-corrected visual acuity (BCVA, logMAR) were recorded during the first clinic visit. Where visual acuity was recorded as counting fingers, a BCVA of 2.1 logMAR was noted; for hand movements, a BCVA of 2.4 logMAR was noted; for light perception, a BCVA of 2.7 logMAR; and for no light perception, a BCVA of 3.0. Clinical diagnosis of every proband was evaluated with thorough ophthalmology examinations and extra-ocular features recorded. The phenotype was determined with color and autofluorescence fundus (AF), optical coherence tomography (OCT), visual evoked potential (VEP), electroretinogram (ERG), visual field (VF), and, when suspected, audiometry. Their clinical blood samples have been in a collection maintained in a −50 °C freezer prior to the NGS experiment. The frozen blood samples were thawed, and the genomic DNA was extracted using the Gentra Puregene Blood kit (Qiagen, USA) according to the manufacturer’s protocol.

Sequencing data have been deposited at NCBI sequence read archive (SRA) (PRJNA952821) and ClinVar. All other data and materials are available from the corresponding authors upon reasonable request.

### IRD gene panel screening

We designed the custom gene panel for the primary screening of IRD, which includes 319 genes associated with IRD (collected from Retnet: https://sph.uth.edu/retnet/ and OMIM: http://www.ncbi.nlm.nih.gov/omim/) (Supplementary Table 2). In addition, the panel also includes 81 noncoding sequences reported for association with IRD. The panel probes were synthesized by IDT (Integrated DNA Technologies, USA), and target capture experiments were conducted in 4 batches of ~200 samples.

For genomic library preps from each sample, the Illumina Nextera Flex for Enrichment kit was applied using 500 ng gDNA and amplified by nine PCR cycles. The individual libraries were quality control (QC) checked by Qubit HS DNA assay (ThermoFisher Scientific, USA) and Fragment Analyzer DNA 6k kit (Agilent, USA) for proper profiles. Then, the libraries were equally pooled and subjected to panel capture according to the Nextera enrichment protocol (Illumina, USA) followed by 12 cycles to amplify the enriched DNA pools. After QC check, the captured DNA pools were sequenced on Illumina HiSeq2500 sequencer (Illumina, USA) to obtain greater than 200-fold coverage per sample.

### Bioinformatics analysis pipeline, variant filtering, in-house BioIT protocol

After short-read sequencing, the Illumina data were mapped and aligned based on GRCh38 (hg38) from the Genome Reference Consortium reference sequence by BWA (bwa-mem). Pipeline output was limited to variants in the target region ±20 bp. First, variants and indels are identified by the joint variant calling pipeline of the Genome Analysis Toolkit (GATK) with HaplotypeCaller and GenotypeVCFs. Then, variant annotation and variant effect prediction are performed with ANNOVAR. After filtering out synonymous SNVs, we removed common SNVs (>1%) based on the frequency in the public database, including those with minor allele frequency (MAF)_ > 0.01 in 1000 G all, 1000 G EAS, ExAC all, ExAC EAS, gnomAD exome all, gnomAD exome EAS, and gnomAD genome all. Finally, variants were classified using a 5-class system consistent with American College of Medical Genetics (ACMG) standards and guidelines for interpreting sequence variants (Supplementary Fig. 1).

The determination of disease-causeative variants is accompanied by an evaluation of three possible modes of genetic inheritance (autosomal recessive, autosomal dominant, and X-linked recessive) based on their pedigree information. This includes examining the sequencing data from affected and unaffected family members to confirm the co-segregation of candidate mutations with the disease. After identifying the putative IRD-associated mutations, Sanger sequencing was performed for predicted class III–V variants to confirm their presence in the study subjects.

### Reporting summary

Further information on research design is available in the Nature Research Reporting Summary linked to this article.

### Supplementary information

Supplementary Information

Supplementary data 1

Supplemtnary Data 2

Supplemtnary Data 3

Supplementary data 4

Reporting Summary

## Data Availability

Sequencing data have been deposited at NCBI Sequence Read Archive (SRA) (PRJNA952821) and ClinVar. All other data and materials are available from the corresponding authors upon reasonable request.

## Code availability

No custom code or scripts were used.
