# Supplementary material for: Highly efficient capture approach for the identification of diverse inherited retinal disorders
Source: NPJ Genom Med. 2024 Jan 9;9:4. doi: 10.1038/s41525-023-00388-3 (PMC10776681; doi:10.1038/s41525-023-00388-3)
Supplement: Supplementary file 1 — Supplementary Information [file 41525_2023_388_MOESM1_ESM.pdf]

Supplementary Information File

Highly efficient capture approach for the identification of diverse inherited retinal disorders

Hsiao-Jung Kao[1*], Ting-Yi Lin[1, 2*], Feng-Jen Hsieh[1], Jia-Ying Chien[3], Erh-Chan Yeh[1], Wan-Jia Lin[1], Yi-Hua Chen[4], Kai-Hsuan Ding[4], Yu Yang[5], Sheng-Chu Chi[6], Ping-Hsing Tsai[5, 7], Chih-Chien Hsu[6, 8], De-Kuang Hwang[6,8] , Hsien-Yang Tsai[9], Mei-Ling Peng[9], Shi-Huang Lee[9], Siu-Fung Chau[9], Chen Yu Chen[9], Wai-Man Cheang[9], Shih-Jen Chen[6, 8], Pui-Yan Kwok[1, 10#], Shih-Hwa Chiou[7, 5, 11#], Mei-Yeh Jade Lu[4#], Shun-Ping Huang[3, 9, 12#]

1. Institute of Biomedical Sciences, Academia Sinica, Taipei, Taiwan
2. Doctoral Degree Program of Translational Medicine, National Yang Ming Chiao Tung University and Academia Sinica, Taiwan
3. Institute of Medical Sciences, Tzu Chi University, Hualien 970, Taiwan
4. Biodiversity Research Center, Academia Sinica, Taipei 11529, Taiwan
5. Department of Medical Research, Taipei Veterans General Hospital, Taipei 11217, Taiwan.
6. Department of Ophthalmology, Taipei Veterans General Hospital, Taipei, Taiwan
7. Institute of Pharmacology, National Yang-Ming University, Taipei 11221, Taiwan
8. School of Medicine, National Yang Ming Chiao Tung University, Taipei, Taiwan
9. Department of Ophthalmology, Taichung Tzu Chi Hospital, Taichung 472, Taiwan
10. Institute for Human Genetics, Cardiovascular Research Institute, and Department of Dermatology, University of California, San Francisco, CA, USA
11. Genomic Research Center, Academia Sinica, Taipei, Taiwan
12. Department of. Molecular Biology and Human Genetics, Tzu Chi University, Hualien 970, Taiwan

*These authors contributed equally, # should be addressed as correspondence

Supplementary Figure legends

Supplementary Figure 1. In-house bioinformatics pipeline

Diagram showing the bioinformatics pipeline. Detailed filtering criteria are delineated in materials and methods. Variants were interpreted in accordance with the American College of Medical Genetics. Pathogenicity classes III, IV, and V were analyzed with the pedigree to perform segregation analysis to obtain the disease-causing variant. Sanger sequencing confirmed the final molecular diagnosis.

Supplementary Figure 2. Worldwide molecular epidemiology of IRD

The world map shows the distribution of the ten most frequent genes reported in the literature. The continental estimated affected individual number is based on Hanany M et al., *PNAS 2020* calculation.

Visual Paradigm Online Free Edition

Supplementary Figure 1

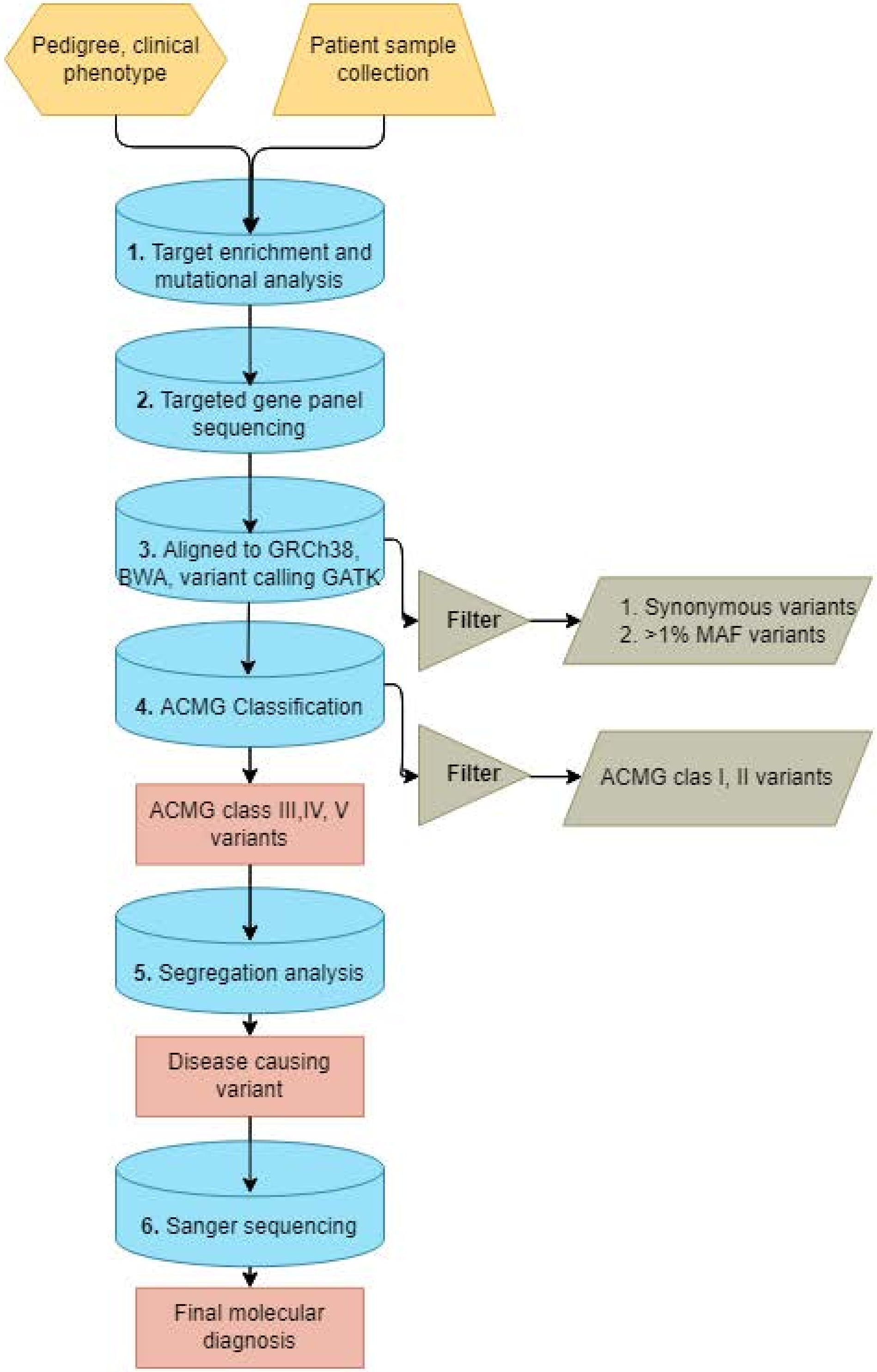

Visual Paradigm Online Free Edition

Supplementary Figure 2

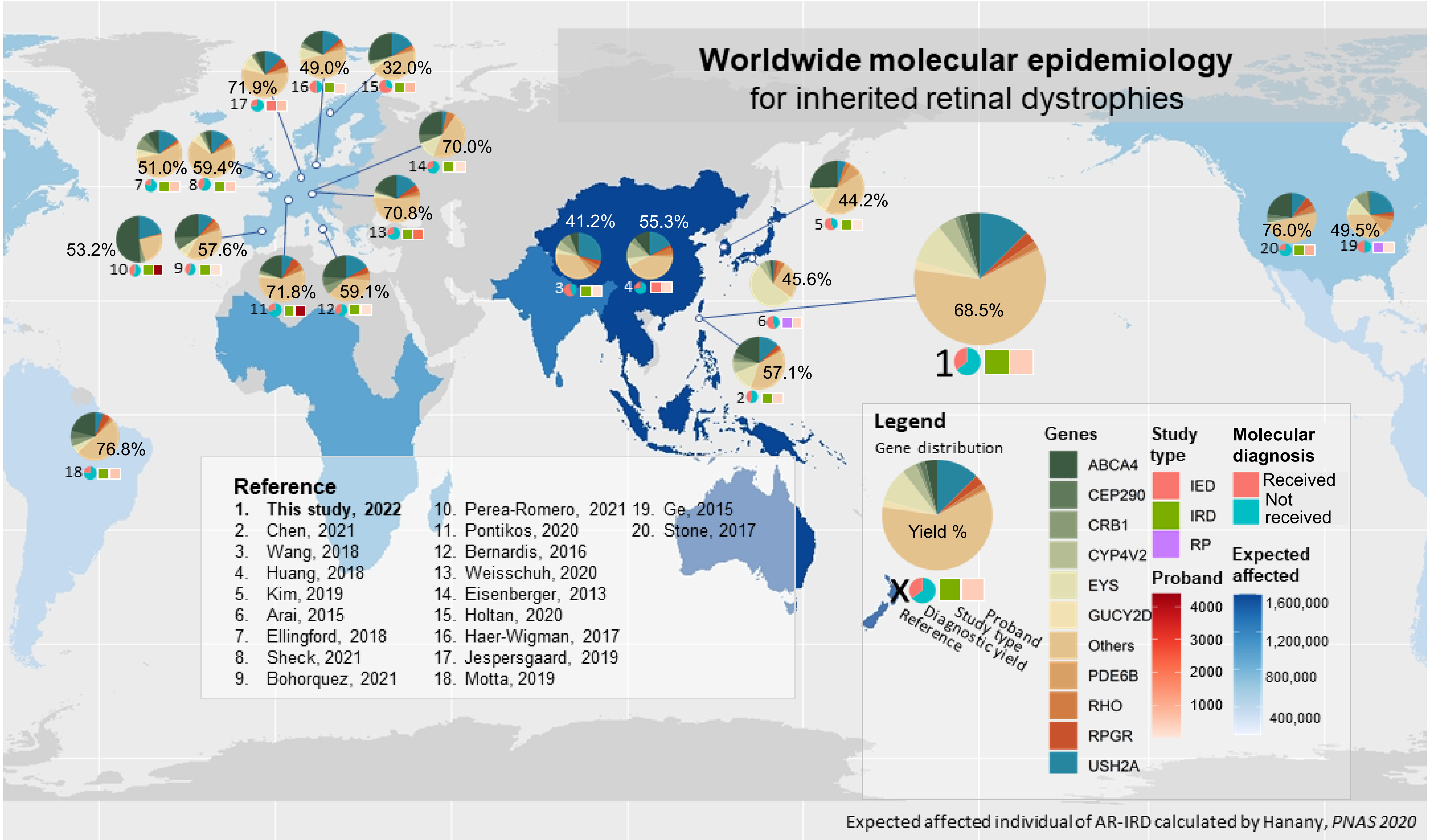

Supplementary Table 1. Clinical trial of current IRD

Available clinical trials of IRD based on gene mutation

IRD	Gene	Sponsors targeting the same gene	Clinicaltrials.gov identifier
Stargardt diseases	*ABCA4*	Sanofi Acucela Alkeus Pharmaceuticals IVERIC Bio Astellas Pharma	NCT01367444 NCT03772665 NCT02402660 NCT03364153 NCT01345006
LCA	*CEP290*	ProQR Therapeutics Editas Medicine/Allergan	NCT03913143 NCT03872479
Choroideremia	*CHM/REP1*	Byron Lam University of Miami Ian M. MacDonald 4D Molecular Therapeutics Biogen STZ eyetrial University of Oxford Spark Therapeutics	NCT02553135 NCT02077361 NCT04483440 NCT03496012 NCT02671539 NCT02407678 NCT02341807
BCD	*CYP4V2*	Beijing Tongren Hospital	NCT04722107
LCA	*GUCY2D*	Atsens Therapeutics Inc	NCT03920007
RP	*MERTK*	King Khaled Eye Specialist Hospital	NCT01482195
RP	*NR2E3*	Ocugen	NCT05203939
RP	*PDE6A*	STZ eyetrail	NCT04611503
RP	*PDE6B*	Horama S.A	NCT03328130
RP	*RHO*	ProQR Therapeutics	NCT04123626
RP	*RLBP1*	Novartis pharmaceuticals	NCT03374657
LCA	*RPE65*	MeiraGTx University of Pennsylvania Applied Genetic Technologies Corp Nantes University Hospital Hadassah Medical Organization Spark Therapeutics	NCT02946879 NCT00481546 NCT00749957 NCT01496040 NCT00821340 NCT00999609
RP	*RPGR*	Applied Genetic Technologies Corp MeiraGTx Nightstar Therapeutics/Biogen	NCT03316560 NCT03252847 NCT03116113
X-linked retinoschisis	*RS1*	Applied Genetic Technologies Corp National Eye Institute (NIH)	NCT02416622 NCT02317887
RP	*USH2A*	ProQR Therapeutics	NCT05158296

Clinical trial citation:

1. Sanofi. Phase I/IIA Study of SAR422459 in Participants With Stargardt's Macular Degeneration Identifier: NCT01367444. Posted June 7, 2011. https://clinicaltrials.gov/ct2/show/NCT01367444
2. Kubota Vision Inc. Safety and Efficacy of Emixustat in Stargardt Disease (SeaSTAR) Identifier: NCT03772665 December 11, 2018 https://clinicaltrials.gov/ct2/show/NCT03772665
3. Alkeus Pharmaceuticals, Inc. Phase 2 Tolerability and Effects of ALK-001 on Stargardt Disease (TEASE) Identifier: NCT02402660 March 30, 2015 https://clinicaltrials.gov/ct2/show/NCT02402660
4. IVERIC bio, Inc. Zimura Compared to Sham in Patients With Autosomal Recessive Stargardt Disease (STGD1) Identifier: NCT03364153 December 6, 2017 https://clinicaltrials.gov/ct2/show/NCT03364153
5. Astellas Pharma Inc Sub-retinal Transplantation of hESC Derived RPE(MA09-hRPE)Cells in Patients With Stargardt's Macular Dystrophy Identifier: NCT01345006 April 29, 2011 https://clinicaltrials.gov/ct2/show/NCT01345006
6. ProQR Therapeutics A Study to Evaluate Efficacy, Safety, Tolerability and Exposure After a Repeat-dose of Sepofarsen (QR-110) in LCA10 (ILLUMINATE) Identifier: NCT03913143 April 12, 2019 https://clinicaltrials.gov/ct2/show/NCT03913143
7. Editas Medicine, Inc. Single Ascending Dose Study in Participants With LCA10 Identifier: NCT03872479 March 13, 2019 https://clinicaltrials.gov/ct2/show/NCT03872479
8. Byron Lam Choroideremia Gene Therapy Clinical Trial Identifier: NCT02553135 September 17, 2015 https://clinicaltrials.gov/ct2/show/NCT02553135
9. University of Alberta An Open Label Clinical Trial of Retinal Gene Therapy for Choroideremia Identifier: NCT02077361 March 4, 2014 https://clinicaltrials.gov/ct2/show/NCT02077361
10. 4D Molecular Therapeutics Dose Escalation Study of Intravitreal 4D-110 in Patients With Choroideremia Identifier: NCT04483440 July 23, 2020 https://clinicaltrials.gov/ct2/show/NCT04483440
11. NightstaRx Ltd, a Biogen Company Efficacy and Safety of BIIB111 for the Treatment of Choroideremia (STAR) Identifier: NCT03496012 April 12, 2018 https://clinicaltrials.gov/ct2/show/NCT03496012
12. STZ eyetrial THOR - Tübingen Choroideremia Gene Therapy Trial (THOR) Identifier: NCT02671539 February 2, 2016 https://clinicaltrials.gov/ct2/show/NCT02671539

13. University of Oxford REP1 Gene Replacement Therapy for Choroideremia (REGENERATE) Identifier: NCT02407678 April 3, 2015 https://clinicaltrials.gov/ct2/show/NCT02407678
14. Spark Therapeutics Safety and Dose Escalation Study of AAV2-hCHM in Subjects With CHM (Choroideremia) Gene Mutations Identifier: NCT02341807 January 19, 2015 https://clinicaltrials.gov/ct2/show/NCT02341807
15. Beijing Tongren Hospital Safety Study of rAAV2/8-hCYP4V2 in Patients With Bietti's Crystalline Dystrophy (BCD) Identifier: NCT04722107 January 25, 2021 https://clinicaltrials.gov/ct2/show/NCT04722107
16. King Khaled Eye Specialist Hospital Trial of Subretinal Injection of (rAAV2-VMD2-hMERTK) Identifier: NCT01482195 November 30, 2011 https://clinicaltrials.gov/ct2/show/NCT01482195
17. QLT Inc. Safety/Proof of Concept Study of Oral QLT091001 in Subjects With Leber Congenital Amaurosis (LCA) or Retinitis Pigmentosa (RP) Due to Retinal Pigment Epithelial 65 Protein (RPE65) or Lecithin:Retinol Acyltransferase (LRAT) Mutations Identifier: NCT01014052 November 16, 2009 https://clinicaltrials.gov/ct2/show/NCT01014052
18. Ocugen The Study to Assess the Safety and Efficacy of OCU400 for Retinitis Pigmentosa and Leber Congenital Amaurosis (OCU400) Identifier: NCT05203939 January 24, 2022 https://clinicaltrials.gov/ct2/show/NCT05203939
19. ProQR Therapeutics A Study to Evaluate the Safety and Tolerability of QR-1123 in Subjects With Autosomal Dominant Retinitis Pigmentosa Due to the P23H Mutation in the RHO Gene (AURORA) Identifier: NCT04123626 October 11, 2019 https://clinicaltrials.gov/ct2/show/NCT04123626
20. MeiraGTx UK II Ltd Long-Term Follow-Up Gene Therapy Study for Leber Congenital Amaurosis OPTIRPE65 (Retinal Dystrophy Associated With Defects in RPE65) Identifier: NCT02946879 October 27, 2016 https://clinicaltrials.gov/ct2/show/NCT02946879
21. University of Pennsylvania Phase I Trial of Gene Vector to Patients With Retinal Disease Due to RPE65 Mutations (LCA) Identifier: NCT00481546 June 1, 2007 https://clinicaltrials.gov/ct2/show/NCT00481546
22. Applied Genetic Technologies Corp Phase 1/2 Safety and Efficacy Study of AAV-RPE65 Vector to Treat Leber Congenital Amaurosis Identifier: NCT00749957 September 10, 2008 https://clinicaltrials.gov/ct2/show/NCT00749957

23. Nantes University Hospital Clinical Gene Therapy Protocol for the Treatment of Retinal Dystrophy Caused by Defects in RPE65 (RPE65) Identifier: NCT01496040 December 21, 2011 https://clinicaltrials.gov/ct2/show/NCT01496040
24. Hadassah Medical Organization Clinical Trial of Gene Therapy for Leber Congenital Amaurosis Caused by RPE65 Mutations Identifier: NCT00821340 January 13, 2009 https://clinicaltrials.gov/ct2/show/NCT00821340
25. Spark Therapeutics Safety and Efficacy Study in Subjects With Leber Congenital Amaurosis Identifier: NCT00999609 October 22, 2009 https://clinicaltrials.gov/ct2/show/NCT00999609
26. Applied Genetic Technologies Corp Safety and Efficacy of rAAV2tYF-GRK1-RPGR in Subjects With X-linked Retinitis Pigmentosa Caused by RPGR Mutations Identifier: NCT03316560 October 20, 2017 https://clinicaltrials.gov/ct2/show/NCT03316560
27. MeiraGTx UK II Ltd Gene Therapy for X-linked Retinitis Pigmentosa (XLRP) - Retinitis Pigmentosa GTPase Regulator (RPGR) Identifier: NCT03252847 August 17, 2017 https://clinicaltrials.gov/ct2/show/NCT03252847
28. NightstaRx Ltd, a Biogen Company A Clinical Trial of Retinal Gene Therapy for X-linked Retinitis Pigmentosa Using BIIB112 (XIRIUS) Identifier: NCT03116113 April 14, 2017 https://clinicaltrials.gov/ct2/show/NCT03116113
29. Applied Genetic Technologies Corp Safety and Efficacy of rAAV-hRS1 in Patients With X-linked Retinoschisis (XLRS) Identifier: NCT02416622 April 15, 2015 https://clinicaltrials.gov/ct2/show/NCT02416622
30. National Eye Institute (NEI) Study of RS1 Ocular Gene Transfer for X-linked Retinoschisis Identifier: NCT02317887 December 17, 2014 https://clinicaltrials.gov/ct2/show/NCT02317887
31. ProQR Therapeutics Study to Evaluate the Efficacy Safety and Tolerability of Ultevursen in Subjects With RP Due to Mutations in Exon 13 of the USH2A Gene (Sirius) Identifier: NCT05158296 December 15, 2021 https://clinicaltrials.gov/ct2/show/NCT05158296

Supplementary Table 2. Panel performance

Performance	Mean	
>Q30 bases (%)	94.2	(0.73)
Total target length (Mbases)	1.2	
Quality Score (std)	37.4	(0.93)
Insert size (std)	205	(12.0)
On target rate (%)	71.1	(2.07)
Near target rate (%)	22.5	(1.66)
Yield (Mbases)	696	(202)
Depth on target region (std)	329	(93.1)
Depth <10 bases (std)	4,660	(32,200)
Depth <20 bases (std)	9,540	(51,600)

The table shows the panel performance in quality control summaries.

Supplementary Table 3. Rate of proband receiving the molecular diagnosis

Onset age	Molecular diagnosis	(%)	ACMG Molecular Diagnosis	(%)
0-10	64/80	80.0	59/80	74.0
10-20	60/86	69.8	44/86	51.2
20-30	46/72	63.9	35/72	48.6
30-40	30/51	58.8	27/51	52.9
40-50	23/37	62.2	19/37	51.4
50-60	17/32	53.1	11/32	34.4
60-70	6/8	75.0	3/8	37.5
70-80	1/2	50.0	0/2	0.00

The table shows the diagnostic yield across age groups.

Supplementary Table 4. Worldwide Molecular Epidemiology ref

Author	Year	Country	Ancestry	Dx rate (%)	Ref
Arai	2015	Japan	East Asian	45.6	[1]
Bernardis	2016	Italy	Southern European	59.1	[2]
Bohorquez	2021	Spanish	Southern European	57.6	[3]
Chen	2021	Taiwan (TIP)	East Asian	57.1	[4]
Eisenberger	2013	Germany	Northwestern European	70	[5]
Ellingford	2016, 2018	UK	Northwestern European	51, 79	[6, 7]
Ge	2015	USA, Canada	European	49.5	[8]
Haer-Wigman	2017	Dutch	Northwestern European	49	[9]
Holtan	2020	Norway	European	32	[10]
Huang(a)	2015, 2018	Chinese	East Asian	55.3, 64.6	[11, 12]
Jespersgaard	2019	Denmark	Northwestern European	71.9	[13]
Kao	2023	Taiwan	East Asian	68.5	This study
Kim	2019	Korea	East Asian	44.2	[14]
Liu	2020, 2021	China	East Asian	44.5, 57	[15, 16]
Motta	2019	Brazil	Latin America	76.8	[17]
Perea-Romero	2021	Spain	Southern European	53.2	[18]
Pontikos	2020	French	Northwestern European	71.8	[19]
Sheck	2021	UK	Northwestern European	59.4	[20]
Stone	2017	USA	European	76	[21]
Wang	2015, 2018	China	East Asian	76.6, 41.2	[22, 23]
Weisschuh	2020	Germany	Northwestern European	70.8	[24]

The table contains the cited literature used to illustrate the worldwide molecular epidemiology of IRD.

Supplementary Data 1. Phenodata *(Excel attachment)*
Female F, male M, family history Fhx, negative N, positive P, visual acuity VA, right eye OD, left eye OS, both eyes OU. Symptomatic S, *Enrolment. VFI: visual field index. VFI 0-30#, 31-60*, 61-80**, 81-100***. N/A, not available. RP: retinitis pigmentosa. ERG: electroretinogram. FAF: fundus autofluorescence. OCT: optical coherence tomography. ONL: outer nuclear layer. EZ: ellipsoid zone. IZ: interdigitation zone. RPE: retinal pigment epithelium. ERM: epiretinal membrane. CME: cystoid macular edema. SRF: subretinal fluid. RD: retinal detachment. VEP: visual evoked potential. VMT: vitreomacular traction. CRT: central retinal thickness. Typical RP phenotypes include bone spicule pigmentation, attenuated retinal vessels, and waxy pallor of the optic disc. Typical BCD triad: glistening yellow-white crystal deposits, tapetoretinal degeneration, and choroidal sclerosis

Supplementary Data 2. Gene panel list *(Excel attachment)*

Selected genes by phenotype used for panel design.

Supplementary Data 3. Genodata *(Excel attachment)*
M, missense; F, frameshift; D, deletion; I, insertion; L, splicing; S, stop gain. The spreadsheet contains two tabs separating variants found in solved cases and unsolved VUS cases.

Supplementary Data 4. Table of previously unreported variants *(Excel attachment)*
The table shows the variant information of unique novel variants. The spreadsheet contains two tabs where in-silico predictions of all variants and in-silico predictions of novel variants are separated.

References

1. Arai, Y., et al., *Retinitis Pigmentosa with EYS Mutations Is the Most Prevalent Inherited Retinal Dystrophy in Japanese Populations.* J Ophthalmol, 2015. **2015**: p. 819760.
2. Bernardis, I., et al., *Unravelling the Complexity of Inherited Retinal Dystrophies Molecular Testing: Added Value of Targeted Next-Generation Sequencing.* Biomed Res Int, 2016. **2016**: p. 6341870.
3. Garcia Bohorquez, B., et al., *Updating the Genetic Landscape of Inherited Retinal Dystrophies.* Front Cell Dev Biol, 2021. **9**: p. 645600.
4. Chen, T.C., et al., *Genetic characteristics and epidemiology of inherited retinal degeneration in Taiwan.* NPJ Genom Med, 2021. **6**(1): p. 16.
5. Eisenberger, T., et al., *Increasing the yield in targeted next-generation sequencing by implicating CNV analysis, non-coding exons and the overall variant load: the example of retinal dystrophies.* PLoS One, 2013. **8**(11): p. e78496.
6. Ellingford, J.M., et al., *Molecular findings from 537 individuals with inherited retinal disease.* J Med Genet, 2016. **53**(11): p. 761-767.
7. Ellingford, J.M., et al., *Assessment of the incorporation of CNV surveillance into gene panel next-generation sequencing testing for inherited retinal diseases.* J Med Genet, 2018. **55**(2): p. 114-121.
8. Ge, Z., et al., *NGS-based Molecular diagnosis of 105 eyeGENE((R)) probands with Retinitis Pigmentosa.* Sci Rep, 2015. **5**: p. 18287.
9. Haer-Wigman, L., et al., *Diagnostic exome sequencing in 266 Dutch patients with visual impairment.* Eur J Hum Genet, 2017. **25**(5): p. 591-599.
10. Holtan, J.P., et al., *Inherited retinal disease in Norway - a characterization of current clinical and genetic knowledge.* Acta Ophthalmol, 2020. **98**(3): p. 286-295.
11. Huang, X.F., et al., *Genotype-phenotype correlation and mutation spectrum in a large cohort of patients with inherited retinal dystrophy revealed by next-generation sequencing.* Genet Med, 2015. **17**(4): p. 271-8.
12. Huang, H., et al., *Systematic evaluation of a targeted gene capture sequencing panel for molecular diagnosis of retinitis pigmentosa.* PLoS One, 2018. **13**(4): p. e0185237.
13. Jespersgaard, C., et al., *Molecular genetic analysis using targeted NGS analysis of 677 individuals with retinal dystrophy.* Sci Rep, 2019. **9**(1): p. 1219.
14. Kim, M.S., et al., *Genetic Mutation Profiles in Korean Patients with Inherited Retinal Diseases.* J Korean Med Sci, 2019. **34**(21): p. e161.
15. Liu, X.Z., Y.Y. Li, and L.P. Yang, *[Comparison study of whole exome*

sequencing and targeted panel sequencing in molecular diagnosis of inherited retinal dystrophies]. Beijing Da Xue Xue Bao Yi Xue Ban, 2020. **52**(5): p. 836-844.
16. Liu, X., et al., *Molecular diagnosis based on comprehensive genetic testing in 800 Chinese families with non-syndromic inherited retinal dystrophies.* Clin Exp Ophthalmol, 2021. **49**(1): p. 46-59.
17. Motta, F.L., et al., *Relative frequency of inherited retinal dystrophies in Brazil.* Sci Rep, 2018. **8**(1): p. 15939.
18. Perea-Romero, I., et al., *Author Correction: Genetic landscape of 6089 inherited retinal dystrophies affected cases in Spain and their therapeutic and extended epidemiological implications.* Sci Rep, 2021. **11**(1): p. 10340.
19. Pontikos, N., et al., *Genetic Basis of Inherited Retinal Disease in a Molecularly Characterized Cohort of More Than 3000 Families from the United Kingdom.* Ophthalmology, 2020. **127**(10): p. 1384-1394.
20. Sheck, L.H.N., et al., *Panel-based genetic testing for inherited retinal disease screening 176 genes.* Mol Genet Genomic Med, 2021. **9**(12): p. e1663.
21. Stone, E.M., et al., *Clinically Focused Molecular Investigation of 1000 Consecutive Families with Inherited Retinal Disease.* Ophthalmology, 2017. **124**(9): p. 1314-1331.
22. Wang, H., et al., *Comprehensive Molecular Diagnosis of a Large Chinese Leber Congenital Amaurosis Cohort.* Invest Ophthalmol Vis Sci, 2015. **56**(6): p. 3642-55.
23. Wang, L., et al., *Application of Whole Exome and Targeted Panel Sequencing in the Clinical Molecular Diagnosis of 319 Chinese Families with Inherited Retinal Dystrophy and Comparison Study.* Genes (Basel), 2018. **9**(7).
24. Weisschuh, N., et al., *Genetic architecture of inherited retinal degeneration in Germany: A large cohort study from a single diagnostic center over a 9-year period.* Hum Mutat, 2020. **41**(9): p. 1514-1527.
