# Supplementary material for: Highly efficient capture approach for the identification of diverse inherited retinal disorders
Source: NPJ Genom Med. 2024 Jan 9;9:4. doi: 10.1038/s41525-023-00388-3 (PMC10776681; doi:10.1038/s41525-023-00388-3)
Supplement: Supplementary file 6 — Reporting Summary [file 41525_2023_388_MOESM6_ESM.pdf]

nature portfolio

Corresponding author(s): PYK, SHC, MYL, SPH

Last updated by author(s): Oct 25, 2023

# Reporting Summary

Nature Portfolio wishes to improve the reproducibility of the work that we publish. This form provides structure for consistency and transparency in reporting. For further information on Nature Portfolio policies, see our Editorial Policies and the Editorial Policy Checklist.

## Statistics

For all statistical analyses, confirm that the following items are present in the figure legend, table legend, main text, or Methods section.

| n/a | Confirmed | |
|---|---|---|
| ☐ | ☒ | The exact sample size (*n*) for each experimental group/condition, given as a discrete number and unit of measurement |
| ☒ | ☐ | A statement on whether measurements were taken from distinct samples or whether the same sample was measured repeatedly |
| ☒ | ☐ | The statistical test(s) used AND whether they are one- or two-sided<br>*Only common tests should be described solely by name; describe more complex techniques in the Methods section.* |
| ☒ | ☐ | A description of all covariates tested |
| ☒ | ☐ | A description of any assumptions or corrections, such as tests of normality and adjustment for multiple comparisons |
| ☒ | ☐ | A full description of the statistical parameters including central tendency (e.g. means) or other basic estimates (e.g. regression coefficient) AND variation (e.g. standard deviation) or associated estimates of uncertainty (e.g. confidence intervals) |
| ☒ | ☐ | For null hypothesis testing, the test statistic (e.g. *F*, *t*, *r*) with confidence intervals, effect sizes, degrees of freedom and *P* value noted<br>*Give P values as exact values whenever suitable.* |
| ☒ | ☐ | For Bayesian analysis, information on the choice of priors and Markov chain Monte Carlo settings |
| ☒ | ☐ | For hierarchical and complex designs, identification of the appropriate level for tests and full reporting of outcomes |
| ☒ | ☐ | Estimates of effect sizes (e.g. Cohen's *d*, Pearson's *r*), indicating how they were calculated |

*Our web collection on statistics for biologists contains articles on many of the points above.*

## Software and code

Policy information about availability of computer code

| | |
|---|---|
| Data collection | No custom code or scripts were used |
| Data analysis | No custom code or scripts were used |

For manuscripts utilizing custom algorithms or software that are central to the research but not yet described in published literature, software must be made available to editors and reviewers. We strongly encourage code deposition in a community repository (e.g. GitHub). See the Nature Portfolio guidelines for submitting code & software for further information.

## Data

Policy information about availability of data

All manuscripts must include a data availability statement. This statement should provide the following information, where applicable:

- Accession codes, unique identifiers, or web links for publicly available datasets
- A description of any restrictions on data availability
- For clinical datasets or third party data, please ensure that the statement adheres to our policy

Sequencing data have been deposited at NCBI Sequence Read Archive (SRA) (PRJNA952821) and ClinVar. All other data and materials are available from the corresponding authors on reasonable request.

## Human research participants

Policy information about studies involving human research participants and Sex and Gender in Research.

| | |
|---|---|
| Reporting on sex and gender | Four hundred ninety-three patients, including 425 probands aged 0 to 96 years with suspected IRD and no previous genetic diagnosis, visited the Department of Ophthalmology at the TVGH and TTCH between January 2018 to May 2022. Age and symptom onset, family history, gender, and best-corrected visual acuity (BCVA, logMAR) was recorded during the first clinic visit. Clinical diagnosis of every proband was evaluated with thorough ophthalmology examinations and extra-ocular features recorded. The phenotype was determined with color and autofluorescence fundus (AF), optical coherence tomography (OCT), visual evoked potential (VEP), electroretinogram (ERG), visual field (VF), and when suspected, audiometry. |
| Population characteristics | See Above. |
| Recruitment | Patients with IRD were recruited from Department of Ophthalmology at Taichung Tzu Chi Hospital and Taipei Veterans General Hospital. |
| Ethics oversight | This study was approved by the Institutional Review Board (IRB) of Academia Sinica (AS-IRB01-21064(N)), Taipei Veterans General Hospital (TVGH, 2021-04-009A), and Taichung Tzu Chi Hospital (TTCH, REC107-24) |

Note that full information on the approval of the study protocol must also be provided in the manuscript.

# Field-specific reporting

Please select the one below that is the best fit for your research. If you are not sure, read the appropriate sections before making your selection.

☒ Life sciences ☐ Behavioural & social sciences ☐ Ecological, evolutionary & environmental sciences

For a reference copy of the document with all sections, see nature.com/documents/nr-reporting-summary-flat.pdf

# Life sciences study design

All studies must disclose on these points even when the disclosure is negative.

| | |
|---|---|
| Sample size | 425 probands with IRD |
| Data exclusions | All probands were included in the data |
| Replication | 425 probands were sequenced, and made molecular diagnoses for 68.5% (291/425) of the probands and 53.9% (229/425) of those consisted of solved diagnosis. RP is the most prevalent IRD (273/425, 64.2%), with the five most common conditions (RP, CRD, US, LCA, and BCD) accounting for 90.8% of the cohort (387/425). Mutations in 5 genes account for 40.8% (119/291) of the molecular diagnoses made: USH2A (40, 13.7%), EYS (33, 11.3%), CYP4V2 (15, 4.8%), ABCA4 (13, 4.5%), RPGR (9, 3.4%), and RP1 (9, 3.1%). Almost half of the positive cases (49.8%) are due to mutations found in the top 10 genes: the 5 genes listed above plus RDH12 (7, 2.4%), CHM (7, 2.4%), ADGRV1 (7, 2.4%), RP2 (6, 2.1%) |
| Randomization | All probands were recruited with clinical diagnosed with IRD. There is no randomization |
| Blinding | All probands were recruited with clinical diagnosed with IRD and all were sequenced using targeted gene sequencing. Blinding is not necessary. |

# Reporting for specific materials, systems and methods

We require information from authors about some types of materials, experimental systems and methods used in many studies. Here, indicate whether each material, system or method listed is relevant to your study. If you are not sure if a list item applies to your research, read the appropriate section before selecting a response.

## Materials & experimental systems

| n/a | Involved in the study |
|---|---|
| ☒ | ☐ Antibodies |
| ☒ | ☐ Eukaryotic cell lines |
| ☒ | ☐ Palaeontology and archaeology |
| ☒ | ☐ Animals and other organisms |
| ☒ | ☐ Clinical data |
| ☒ | ☐ Dual use research of concern |

## Methods

| n/a | Involved in the study |
|---|---|
| ☒ | ☐ ChIP-seq |
| ☒ | ☐ Flow cytometry |
| ☒ | ☐ MRI-based neuroimaging |
